# Associations between genetic variations in the FURIN gene and hypertension

**DOI:** 10.1186/1471-2350-11-124

**Published:** 2010-08-13

**Authors:** Nanfang Li, Wenli Luo, Zhang Juhong, Jin Yang, Hongmei Wang, Ling Zhou, Jianhang Chang

**Affiliations:** 1 The Center of Diagnosis, Treatment and Research of Hypertension in Xinjiang, Urumqi, Xinjiang 830001, China

## Abstract

**Background:**

Hypertension is a complex disease influenced by multiple genetic and environmental factors. The Kazakh ethnic group is characterized by a relatively high prevalence of hypertension. Previous research indicates that the FURIN gene may play a pivotal role in the renin-angiotensin system and maintaining the sodium-electrolyte balance. Because these systems influence blood pressure regulation, we considered FURIN as a candidate gene for hypertension. The purpose of this study was to systematically investigate the association between genetic variations in the FURIN gene and essential hypertension in a Xinjiang Kazakh population.

**Methods:**

We sequenced all exons and the promoter regions of the FURIN gene in 94 hypertensive individuals to identify genetic variations associated with the disorder. Genotyping was performed using the TaqMan polymerase chain reaction method for four representative common single nucleotide polymorphisms (SNPs, -7315C > T, 1970C > G, 5604C > G, 6262C > T) in 934 Kazakh Chinese people. One SNP (1970C > G) was replicated in 1,219 Uygur Chinese people.

**Results:**

Nine novel and seven known single nucleotide polymorphisms were identified in the FURIN gene. The results suggest that 1970C > G was associated with a hypertension phenotype in Kazakh Chinese (additive model, *P *= 0.091; dominant model, *P = *0.031, allele model, *P *= 0.030), and after adjustment with logistic regression analysis, ORs were 1.451 (95%CI 1.106-1.905, *P *= 0.008) and 1.496 (95% 1.103-2.028, *P *= 0.01) in additive and dominant models, respectively. In addition, the association between 1970C > G and hypertension was replicated in Uygur subjects (additive model, *P *= 0.042; dominant model, *P *= 0.102; allele model, *P *= 0.027) after adjustment in additive and dominant models, ORs were 1.327 (95% 1.07-1.646), *P *= 0.01 and 1.307 (95%CI 1.015-1.681, *P *= 0.038), respectively. G allele carriers exhibited significant lower urinary Na^+ ^excretion rate than non-carriers in the Kazakh Chinese population (152.45 ± 76.04 uM/min vs 173.33 ± 90.02 uM/min, *P *= 0.007).

**Conclusion:**

Our results suggest that the FURIN gene may be a candidate gene involved in human hypertension, and that the G allele of 1970C > G may be a modest risk factor for hypertension in Xinjiang Kazakh and Uygur populations.

## Background

Essential hypertension is considered a typical complex disease with a multifactorial etiology, which has led to inconsistent findings in genetic studies. A body of evidence suggests that the renin-angiotensin system (RAS) and sodium-electrolyte balance play a pivotal role in the development and progression of hypertension.

A receptor that is specific for renin and prorenin, named the (pro) renin receptor ([P] RR), was first identified in human mesangial cells [1]. Studies to date have focused on the functional aspects of the protein, because of the potential role of (P) RR in hypertension and organ damage [2]. FURIN is an important enzyme participating in (P) RR processing. Recently, Nguyen et al demonstrated that FURIN, as a protease, can shed endogenous (P)RR [3]. Colon carcinoma cells devoid of active FURIN were found to synthesize full-length (P)RR, but not to secrete s(P)RR. Transfection of Chinese hamster ovary cells with a plasmid coding for the a1-antitrypsin Portland variant, an inhibitor of FURIN, completely inhibited the generation of s(P)RR, whereas the addition in the culture medium of GM6001, an inhibitor of metalloproteases, or TNF-a protease inhibitor-1, an inhibitor of ADAM17, had no effect. When the cDNA coding for (P)RR was translated *in vitro *and incubated with recombinant FURIN or ADAM17, only FURIN was found to generate the 28 kDa-s(P)RR. Moreover, mutagenesis at the potential FURIN cleavage R275A/KT/R278A site abolished s(P)RR generation [3].

In addition, it has been well reported that the epithelial Na+ channel (ENaC) is critical for Na^+ ^homeostasis and blood pressure (BP) control [4], and that defects in ENaC function and genetic structure can cause inherited forms of hypertension and essential hypertension [5,6]. Endothelin may participate in blood-pressure elevation and vascular growth in moderate to severe hypertension [7]. Furthermore, it is well established that the TGF-β signaling pathway has a role in BP homeostasis [8]. Therefore, factors that simultaneously participate in the regulation of function of these genes are expected to be associated with hypertension. The proprotein processing enzyme FURIN is the mammalian prototype of a novel family of subtilisin-like serine endoproteases which possess cleavage specificity for sites involving multiple basic amino acid residues and are involved in the processing of precursor proteins of a variety of regulatory peptides and proteins [9,10]. Recent work suggests that ENaC is synthesized and transported from the endoplasmic reticulum to the Golgi apparatus in an inactive form. In the Golgi apparatus, FURIN proteolytically cleaves specific sites in the extracellular domains of the a-and γ-subunits, and this cleavage appears to activate ENaC [11]. In addition, proendothelin-1 is subjected to proteolysis at specific pairs of basic amino acids by FURIN, which may also participate in the maturation of proendothelin-1 in endothelial cells [12]. Latent TGF-β exhibits an appropriate R-H-H-R cleavage motif and is, consequently activated by FURIN [13-15]. Zacchigna et al. reported that the extracellular protein Emilin1 inhibits TGF-β signaling by binding specifically to the proTGF-β precursor and preventing its maturation by FURIN in convertases and Emilin1 knockout animals display increased BP [16].

The human FURIN gene, consisting of sixteen exons and fifteen introns that encode 795 amino acid residues, is located on chromosome 15q26.1, where several loci have shown strong or suggestive linkage to BP and related phenotypes by genome-wide linkage scans [17]. As such, the human FURIN gene is a candidate gene potentially underlying BP elevation.

The Xinjiang Kazakh are an ethnic minority group with the highest prevalence of hypertension in China, exhibiting an age-standardized prevalence of 39.8% [18]. A previous study reported that, compared with other Chinese minority ethnic groups in Xinjiang, the Kazakh population has a higher average salt consumption, with a mean daily consumption of > 21 g. BP has been found to decrease significantly after limiting salt intake [18,19]. As such, the Kazakh population is ideal for studying the genetic factors involved in salt-sensitive hypertension. So far, there are no reports about the relationship between genetic variations in the human FURIN gene and hypertension, or the genotyping of their representative variations in the general population. To address this issue, we systemically investigated the association between variations of the FURIN gene, hypertension and BP in a Xinjiang Kazakh population.

## Methods

### Subjects

A total of 1,000 Kazakh subjects, with no miscegenation, were randomly recruited for this study by multistage cluster sampling from the Fukang area in the Xinjiang Uygur Autonomous Region. Subjects with a history of secondary hypertension, stroke, excessive drinking, cancer and use of contraceptives were excluded from this study. After these exclusions, 934 participants went through the survey during a one-month period from January to February 2008. Written consent was obtained from all subjects before any data collection and measurement. Subjects were divided into two groups: a hypertensive group and normotensive group. We adopted the WHO's 1999 definition of hypertension in this study: a systolic blood pressure (SBP) of at least 140 mmHg and/or diastolic blood pressure (DBP) of at least 90 mmHg or anti-hypertension treatment. Non-hypertensive participants met the following criteria: no history of any antihypertensive medications, and SBP of less than 140 mmHg and DBP of less than 90 mmHg. Diabetes mellitus (DM) was defined by: a fasting plasma glucose level of 126 mg/dL or greater (≥ 7.0 mmol/L), or a 2-hour postchallenge plasma glucose level of 200 mg/dL or greater (≥ 11.1 mmol/L) or the current use of antidiabetic medication. Hyperlipidemia was defined as total cholesterol ≥ 220 mg/dl or triglyceride ≥ 150 mg/dl, or the current use of antihyperlipidemia medication. BP was measured three times in the left arm with a standard sphygmomanometer while the subject was seated after at least 10 minutes of rest, BP measurements were taken twice in the continuous two days. Study subjects underwent routine laboratory tests, including serum and urinary electrolytes, renal function, blood glucose, total cholesterol and triglyceride. The urinary Na^+ ^excretion rate (UNa^+ ^rate) was calculated as UNa*UV/24 h, where UNa* was the urinary concentration of Na^+^, and UV was the urinary volume in 24 h. All clinical data, sequencing and genotyping results were performed anonymously. The study was approved by the Ethnics Committee of the People's Hospital, Xinjiang.

### Screening of genetic variations in the FURIN gene in hypertension patients

We sequenced all exons and the promoter region of the FURIN gene. Blood samples were obtained from 94 hypertensive patients, who were randomly chosen from the hypertension group of the study population, and genomic DNA was isolated from peripheral blood leukocyte by using PAXgene Blood DNA Kit (PreAnalytiX™). All exons with their flanking sequences and approximately 1.0 kb of the upstream region were directly sequenced using an ABI PRISM 3700 DNA analyzer (Applied Biosystems, Foster City, California, USA) using twenty sets of primers as described previously [20] (see additional file 1). The obtained sequences were examined for the presence of variations using Sequencher 4.7 software (Gene Codes Corporation, Ann Arbor, Michigan, USA), followed by visual inspection. The A of the ATG of the initiator Met codon is denoted as nucleotide + 1. A GenBank nucleotide sequence (accession ID NT-010274) was used as a reference sequence.

### Genotyping of representative single nucleotide polymorphisms (SNPs) in the general population

After studying the linkage disequilibrium (LD) among SNPs, four selected representative common SNPs, with a minor allele frequency greater than 10%, were used to genotype DNA samples from the study cohort. TaqMan SNP Genotyping Assays were performed for genotyping using the Taq amplification method in a 7900 HT Fast Real-Time PCR system (Applied Biosystems, USA). Five μl PCR reactions were performed using 1 μl DNA (25 ng/μl) or H_2_O for negative controls, 0.125 μl probe (20 μmol/L), and 2.25 μl Mast Mix (20 μmol/l; Applied Biosystems, USA). The reaction was cycled 43 times with a denaturation step of 95°C for 10 seconds, an annealing step of 60°C for 1 minute, and a final elongation step of 72°C for 7 minutes. The primers and probes (Applied Biosystems) used in the TaqMan SNP Genotyping Assays were chosen based on information available on the Applied Biosystems Inc.website http://myscience.appliedbiosystems.com. All of the four selected representative SNPs were successfully genotyped in 925 subjects. For genotyping quality control, the case and control subjects were distributed randomly across the plates, and the samples sequenced also were genotyped for detecting genotyping errors. The call rate for genotyping was 98.61% and the concordance of duplicates was 100%.

Values are expressed as means ± SD. The distribution of patient characteristics or genotypes or allele frequency of SNPs between normotensive and hypertensive groups was analyzed using Student's *t*- tests or *χ*^*2 *^analysis. The associations of four common SNPs with hypertension were analyzed using logistic regression analysis, adjusting for age and gender. All analyses were performed using the SPSS15.0. Statistical significance was established at *P *< 0.05. LD and Hardy-Weinberg equilibrium were analyzed using the SNPAlyze version 2.1 (DYNACOM Co. Ltd., Mobara, Japan).

## Results

### Study Population

The characteristics of the Kazakh and Uygur populations are summarized in Table 1, sorted into hypertensive and normotensive groups. There were significant differences between hypertensive and normotensive groups in some variables, including age, BMI, SBP, DBP, and prevalence of DM, but no significant differences were found for gender and HDL-cholesterol between the two groups in our two populations.

**Table 1:** Basic characteristics of subjects in hypertensives and normotensives

Characteristics	Kazakh Chinese		Replication(Uygur Chinese)	
	NT	HT	NT	HT
Gender (men/women, n)	193/292	211/238**	165/309	261/436**
Age (years)	41.97 ± 7.44	48.40 ± 7.45*	48.03 ± 11.07	52.19 ± 10.43*
BMI (Kg/m2)	25.05 ± 3.48	28.68 ± 4.40*	26.39 ± 4.28	27.40 ± 4.56*
SBP (mmHg)	114.61 ± 8.92	165.05 ± 22.88*	111.37 ± 11.52	142.86 ± 28.35*
DBP (mmHg)	76.13 ± 6.27	104.63 ± 12.05*	69.19 ± 8.7	84.83 ± 16.76*
Total cholesterol (mmol/L)	4.88 ± 1.04	5.12 ± 1.01*	4.33 ± 1.19	4.55 ± 1.24*
HDL-cholesterol (mmol/L)	1.47 ± 0.39	1.46 ± 0.43*	1.09 ± 0.34	1.09 ± 0.32
LDL-cholesterol (mmol/L)	2.92 ± 0.79	3.11 ± .079*	2.41 ± 1.04	2.63 ± 1.12*
Triglyceride (mmol/L)	0.99 ± 0.55	1.30 ± 0.83*	1.43 ± 0.89	1.72 ± 1.43*
Fasting glucose (mmol/L)	4.85 ± 0.56	5.42 ± 0.69*	5.42 ± 1.84	6.0 ± 2.79*
Diabetes mellitus(%)	4.90	2.01**	14.9	25.1**

NT, normotensives; HT, hypertensives; BMI, body mass index; SBP, systolic blood pressure; DBP, diatolic blood pressure. Data are mean ± std or percentage. *P *values were analyzed using Student's *t*- test. Diabetes, fasting plasma glucose 6.0 mmol/l or greater or non-fasting plasma glucose level of 11.1 mmol/l or greater, or antidiabetic Medication.;*P < 0.05 between normotensives and hypertensives by t-test; **P < 0.05 between normotensives and hypertensives by χ^2 ^test.

### Identification of Polymorphisms and selection of representative SNPs in FURIN

Direct sequencing using DNA samples from 94 hypertension subjects revealed sixteen SNPs in the FURIN gene, including two SNPs in the promoter region, one SNP in the 5'-untranslated region, five synonymous mutations in exon 13 and exon 16, four SNPs in the 3'-untranslated region, and five SNPs in introns (Table 2). Among these SNPs, there were six common SNPs with a minor allele frequency over 0.05. No missense or frameshift mutations were identified. Three SNPs, -7315C > T, 4573G > T and 6262C > T, and another three SNPs, 1970C > G, 2003T > C, and 6262C > T, were in LD (*r*^2 ^> 0.5), respectively. Finally, four common SNPs, -7315C > T in the promoter region, 1970C > G in intron 7, 5604C > G in exon 16 and 6262C > T in the 3'-untranslated region, were selected for genotyping.

**Table 2:** Sequence variations in the promoter region and exons in Furin identified in Kazakhs hypertensive patients

SNP name	LD	Region	Aminoacid substitution	Allele 1 freq	Allele 2 freq	Flanking sequence	Typing	db SNP ID
-7393C > G		promoter		0.9884	0.0116	acacaaggag[c/g]tggagctggc		
-7315C > A	a	promoter		0.7733	0.2267	aagtgcagac[c/a]caccccaata	TaqMan	rs4932178
-7048T > C		5'-UTR		0.9942	0.0057	tcccaggtgc[t/c]ctggagctgg		
1970C > G	b	intron7		0.8046	0.1954	gccactttcc[c/g]actgtggatc	TaqMan	rs2071410
2000G > C		intron7		0.9942	0.0058	gaaagagctg[g/c]acccctgtgg		
2003T > C	b	intron7		0.8081	0.1919	ggctgttcca[t/c]ggagggttcc		rs1573643
2589C > T		intron8		0.9945	0.0055	tgtccagccc[c/t]tgcgggcagg		
4369G > T		exon13	R464R	0.9940	0.0060	tcgggaaacg[g/t]ctcgaggtgc		rs6225
4393C > T		exon13	T472T	0.9759	0.0241	agaccgtgac[c/t]gcgtgcctgg		
4573G > T	a	intron13		0.7590	0.2410	cagcgccgcc[g/t]cctctcacag		rs6224
5604G > C		exon16	G617G	0.4048	0.5952	gccctccagg[g/c]ttcgcccccc	TaqMan	rs6226
6123C > T		exon16	D770D	0.9943	0.0057	ttatcaaaga[c/t]cagagcgccc		
6262C > T	a	3'-UTR		0.7989	0.2011	gtggagactg[c/t]ttcccatcct	TaqMan	rs6227
6563G > T		3'-UTR		0.9943	0.0057	tgagggagga[g/t]gccacctctc		
6624C > T		3'-UTR		0.9886	0.0114	tgagtcttgg[c/t]ggcagcagcc		
6728C > T		3'-UTR		0.9943	0.0057	ccctgtgctc[c/t]gtgcctccac		

The apparent linkage disequilibrium (LD), defined by r-square more than 0.5, was indicated by a-b in the LD column. Taqman, The single nucleotide polymorphism (SNP) was successfully genotyped by the Taqman method. UTR, Untranslated region. The A of the ATG of the initiator Met codon is denoted nucleotide +1, as recommended by the Nomenclature Working Group (Hum Mut 1998; 11:1-3). The nucleotide sequence (GenBank Accession ID: NT-010274) was used as a reference sequence.

### Association of the four representative common SNPs with hypertension

Genotype frequencies of the four SNPs (-7315C > T, 1970C > G, 5604C > G, and 6262C > T) satisfied the Hardy-Weinberg equilibrium both in the hypertensive group and in the normotensive group (P > 0.05). Table 3 shows the genotype distributions of the four representative common SNPs between hypertensive and normotensive groups and the replication of 1970C > G in the Uygur population.

The present study did not find any evidence for an association between -7315C > T, 5604C > G or 6262C > T and hypertension. However, 1970C > G was significantly associated with the hypertension phenotype in Kazakh Chinese in a dominant model (*P = *0.031), but not in an additive model (*P *= 0.091). However, after adjustment for age, gender and present ills (diabetes mellitus and dyslipidemia) in multivariate logistic regression analysis, ORs were 1.451(95% CI 1.106-1.905), *P *= 0.008 in an additive model and 1.496(95%CI, 1.103-2.028), *P *= 0.01 in a dominant model. Data are shown in Table 3. In an allele model, the allelic frequencies of C and G were 87.37% (844) and 12.63% (122) in normotensives, respectively; and 83.82% (741) and 16.18% (143) in hypertensives, respectively. Hypertensives exhibited a significantly higher allelic frequency of G (χ^2 ^= 4.732, *P *= 0.030; data not shown).

**Table 3:** Distribution of Genotypes for Furin Variable in Hypertensives and Normotensives

Population	Variants	Genotypes	NT	HT	Additive model	**χ**^**2**^	*P*
			n(%)	n(%)	OR(95%CI)		
		CC	315(65.6)	290(65.3)			
		CT	143(29.8)	134(30.2)	1.1 (0.87-1.391)	0.018	0.991*
	-7315C > T	TT	22(4.8)	20(4.5)			0.424†
	rs4932178						
		CC CT+TT	315(65.6)	290(65.3)		0.01	0.921*
			165(34.6)	154 (34.7)	1.132 (0.853-1.503)		0.391†
							
		GG	171(35.7)	145(30.27)			
	5604G > C	GC	239(49.9)	219(45.72)	1.084 (0.891-1.319)	2.286	0.319*
	rs6226	CC	69(14.41)	79(16.49)			0.42†
							
Kazakh Chinese		CG CG+GG	171(35.7)	145(30.27)	1.031 (0.776-1.369)	0.9	0.343*
			308(50.1)	298(46.76)			0.834†
							
		CC	362(74.9)	322(72)			
	6262C > T	CT	113(23.4)	113(25.3)	1.284 (0.981-1.681)	1.748	0.417*
	rs6227	TT	8(1.7)	12(2.7)			0.69†
							
		CT vs CT+TT	362(74.9)	322(72)	1.28 (0.945-1.733)	1.012	0.313*
			121(25.1)	125(28)			0.111†
							
		CC	369(76.4)	310(45.7)			
	1970C > G	CG	106(21.9)	121(27.4)	1.451 (1.106-1.905)	4.784	0.091*
	rs2071410	GG	8(1.7)	11(2.5)			0.008†
		CG vs CG+GG	369(76.4)	310(45.7)	1.496 (1.103-2.028)	4.636	0.031*
			114(22.6)	132(29.9)			0.01†
							
		CC	322(67.9)	472(63.4)			
Replication (Uygur Chinese)	1970C > G	CG	140(29.5)	234(31.4)	1.327 (1.07-1.646)	6.323	0.042*
	rs2071410	GG	12(2.5)	39(5.2)			0.01†
		CG vs CG+GG	322(67.9)	472(63.4)	1.307 (1.015-1.681)	2.672	0.102*
			160(32)	273(36.6)			0.038†

NT, normotensives; HT, hypertensives. In the additive model, odds ratios (ORs) were expressed per difference in number of minor alleles. In the dominant model, ORs were shown as heterozygotes and minor allele homozygotes compared with major allele homozygotes.*P was caculated by χ^2 ^test; †*P *and ORs were caculated by mutivariable logistic regression analysis after adjustment for gender, age and present ills(dibetes mellitus and dyslipidemia)

In addition, we also examined hypertension and normotension, defined as ≥160/100 mmHg and ≤130/80 mmHg, respectively. We found a significant association between 1970C > G and hypertension (ORs were 1.653[95% CI 1.1106-2.462; *P *= 0.013] in an additive model and 1.854[95%CI, 1.193-2.881; *P *= 0.006] in a dominant model). Data are shown in table 4.

**Table 4:** Distribution of Genotypes for 1970C > G in subgroups in Kazakh Chinese

Polymorphisms	Genotypes	Subgroup1	Subgroup 2	OR(95%CI)	**χ**^**2**^	*P*
		n(%)	n(%)			
1970C > G	CC	311(76.0)	112(67.5)		5.715	0.057*
	CG	90(22.0)	52(31.3)	1.653 (1.11-2.462)		0.013†
(rs2071410)	GG	8(2.0)	2(1.2)			
	CC	311(76.0)	112(67.5)	1.854 (1.193-2.881)	4.459	0.035*
	CG+GG	98(24.0)	52(32.5)			0.006†

Subgroup 1, defined as systolic blood pressure ≤ 130 mmHg, and diabolic blood pressure ≤ 80 mmHg; Subgroup 2, defined as systolic blood pressure ≥ 160 mmHg, and diabolic blood pressure ≥ 100 mmHg. *, calculated by χ^2 ^test; †, ORs are calculated by Logistic regression analysis.

### Repetition

Genotyping with the TaqMan polymerase chain reaction method was performed for one common SNP (1970C > G) in the general Uygur population, with a sample size of 1,219 individuals (745 hypertensives and 474 normotensives). The distribution of the genotype was significantly different between hypertensives and normotensives in an additive model (*P *= 0.042), and after adjustment for age and gender in logistic regression analysis, the OR was 1.327 (95%CI 1.07-1.646; *P *= 0.01). Although in a dominant model, the genotype distribution was not significantly different (*P *= 0.102), the adjusted OR was 1.307(95%CI 1.015-1.681), *P *= 0.038. In an allele model, the hypertensive group had a significantly higher allelic frequency of G (χ^2 ^= 4.886, *P *= 0.027). This finding is consistent with the results found in the Kazakh population.

### Intermediate phenotype

Table 5 presents the comparison of the Urinary Na^+ ^excretion rate between different genotypes by covariate variance analysis, adjusting for gender, age and present ills (diabetes mellitus and dyslipidemia). For Kazakh Chinese, the Urinary Na^+ ^excretion rate was significantly lower for CC than CC+CT individuals (*P *= 0.007). However, there was no significant difference between genotypes in the Uygur Chinese population (*P *= 0.464).

**Table 5:** Comparing of urinary Na+ excretion rate between different genotypes

	Population	1970C > G		*P value*
			
		non-carrier	carrier	
UNa^+ ^rate (uM/min)		n = 549	n = 195	
	Kazakh Chinese	173.33 ± 90.02	152.45 ± 76.04	0.007*
		n = 599	n = 308	
	Uygur Chinese	140.21 ± 79.80	144.52 ± 89.10	0.464*

UNa^+ ^rate, Urinary Na+ excretion rate; All the skew datas were transformed by logarithm. *P was calculated by Covariate variance analysis after adjustment for gender age and present ills(diabetes mellitus and dyslipidemia).

## Discussion

The present study is the first to examine the relationships between genetic variations in the FURIN gene and hypertension in humans. By systemically screening variations of FURIN and studying the associations of four representative common SNPs with hypertension, and we did not identify any rare functional mutations in the functional regions of FURIN. However, the data indicated possible associations between the common SNP 1970C > G and hypertension, and this was replicated in the general Uygur population. These findings indicate that the FURIN gene may be a candidate gene involved in human hypertension, and that G allele of 1970C > G may be a modest risk factor for hypertension.

Hypertension, as a complex trait, has been suggested to be caused by common sequence variants that may have a small to moderate phenotypic effect [21,22]. On the other hand, some studies have shown that most Mendelian disorders are caused by a set of different rare mutations that reside in coding regions, which tend to have strong phenotypic effects. Several recent studies have shown that rare genetic variations in ABCA1, APOA1, and LCAT, collectively contribute to variation in plasma levels of HDL-cholesterol in the general population [23-25]. As such, we hypothesize that both common rare genetic variations in the FURIN gene may contribute to hypertension. In this study, we systemically investigated the association between genetic variations of the FURIN gene and hypertension in a Xinjiang Kazakh population by screening genetic variations of the FURIN gene, then examining the relations between the representative SNPs and hypertension. This research strategy was selected for the following reasons: a) HapMap project does not provide genetic information for Xinjiang Kazakhs. In the HapMap database, the minor allelic frequencies of rs4932178, rs6226, rs6227 and rs2071410 are different among different ethnic groups (CEU:38.5%, 0%, 38.3% and 39%; CHB:16.7%, 5%, 4.40% and 4.44%; JPT: 14%, 4%, 9.1% and 10.2%; YRI:32.7%, 0%, 26.7% and 14.2%, compared to Xinjiang Kazakh [from the present results]: 19.53%, 40.89%, 14.30% and 14.32%, respectively). Thus, the genotype frequencies of common SNPs in the Kazakh population differ from those in the other ethnic groups. As such, we could not use the Tag-SNP specific for Kazakhs in this study. b) By sequencing the functional regions of the FURIN gene in 94 Kazakh hypertensives, we were able to find common and rare SNPs or mutations, both of which are considered to contribute to the pathogenesis of hypertension.

In this study, we did not find any missense/frameshift mutations in the FURIN gene. This may be indicative of the relatively high conservation of the FURIN gene and the importance of this molecule in regulating BP. A significant association between the common SNP 1970C > G and hypertension was obtained in our multivariable analysis with adjustment for age and gender. In the present study, we genotyped four SNPs that are not in a tight LD. Thus, it is necessary to perform a correction for multiple testing. After correcting with the Bonferroni method, no SNPs were significantly associated with hypertension. However, a possible association of 1970C > G with hypertension was re-confirmed in the general Xinjiang Uygur population, and the G allele carriers exhibited a significantly lower urinary Na^+ ^excretion rate in Kazakh Chinese people. Because the Kazakh population consumes an extremely high amount of salt, we defined hypertension and normotension as ≥ 160/100 mmHg and ≤ 130/80 mmHg, respectively, to reduce the possibility of misclassification. A significant association remained between 1970C > G and hypertension in subgroups (*P *< 0.05). Moreover, distribution of all polymorphisms conformed to the Hardy-Weinberg equilibrium, suggesting that the results of this study are unlikely to have been biased by population stratification or admixture for essential hypertension. All of these findings indicate that our results are likely to be reliable.

The mechanisms by which the common SNP 1970C > G might contribute to hypertension are currently unknown. 1970C > G is located in an intronic region. It has traditionally been assumed that the sequence of any given intron is junk DNA with no biological function. More recently, however, this notion has been disputed [26]. Introns contain several short sequences that are important for efficient splicing, such as acceptor and donor sites at either end of the intron as well as a branch point site, which are required for proper splicing by the spliceosome. Therefore, further investigation is necessary to determine whether the polymorphisms of 1970C > G affect the splicing of mRNA of the FURIN gene. In addition, it remains possible that 1970C > G is a mere genetic marker, and it may be that LD with other functional variations within the FURIN gene and other functional polymorphisms play more important roles in hypertension. A greater density of genotyping around the FURIN gene or 1970C > G site is needed to address these issues.

## Conclusion

In summary, our results indicate that the G allele of 1970C > G in the FURIN gene may be an independent risk factor for hypertension. We propose that genetic variations of the FURIN gene collectively contribute to the pathogenesis of hypertension in the Xinjiang Kazakh and Uygur populations.

## Competing interests

The authors declare that they have no competing interests.

## Authors' contributions

NFL, JY WLL and JHZ drafted the paper; WLL, JHZ, HMW and JHC performed experiments; JHZ and LZ performed the statistical analysis; NFL conceived of the study, participated in its design and coordination. All authors read and approved the final manuscript.

## Pre-publication history

The pre-publication history for this paper can be accessed here:

http://www.biomedcentral.com/1471-2350/11/124/prepub

## Supplementary Material

Additional file 1 **Primers for polymerase chain reaction (PCR) and sequencing for Furin gene**. Aditional file 1 showed the information on primers for polymerase chain reaction and sequencing Click here for file
