# Supplementary material for: Associations between genetic variations in the FURIN gene and hypertension
Source: BMC Med Genet. 2010 Aug 13;11:124. doi: 10.1186/1471-2350-11-124 (PMC2936893; doi:10.1186/1471-2350-11-124)
Supplement: Additional file 1 — Primers for polymerase chain reaction (PCR) and sequencing for Furin gene. Aditional file 1 showed the information on primers for polymerase chain reaction and sequencing [file 1471-2350-11-124-S1.DOC]

**Supplementary table 1** Primers for polymerase chain reaction (PCR) and sequencing for furin gene
